# Supplementary material for: Crystal Structure-Based Exploration of Arginine-Containing Peptide Binding in the ADP-Ribosyltransferase Domain of the Type III Effector XopAI Protein
Source: Int J Mol Sci. 2019 Oct 14;20(20):5085. doi: 10.3390/ijms20205085 (PMC6829252; doi:10.3390/ijms20205085)
Supplement: Supplementary file 1 [file ijms-20-05085-s001.pdf]

Article

Crystal structure-based exploration of arginine-containing peptide binding in the ADP-ribosyltransferase domain of the type III effector XopAI protein

Jyung-Hurng Liu [1,2,3,4,*], Jun-Yi Yang [3,5], Duen-Wei Hsu [6], Yi-Hua Lai [2], Yun-Pei Li [1], Yi-Rung Tsai [1], and Ming-Hon Hou [1,2,3,4]

1 Institute of Genomics and Bioinformatics, National Chung Hsing University (NCHU), Taichung, Taiwan 40227, R.O.C.; jhliu@nchu.edu.tw (J.-H.L.); yunk293@gmail.com (Y.-P.L.); stave0972@gmail.com (Y.-R.T.); mhho@nchu.edu.tw (M.-H.H.)
2 Department of Life Science, NCHU, Taichung, Taiwan 40227, R.O.C.; jhliu@nchu.edu.tw (J.-H.L.); hbm486426@gmail.com (Y.-H.L.); mhho@nchu.edu.tw (M.-H.H.)
3 Graduate Institute of Biotechnology, NCHU, Taichung, Taiwan 40227, R.O.C.; jhliu@nchu.edu.tw (J.-H.L.); jyang@nchu.edu.tw (J.-Y.Y.); mhho@nchu.edu.tw (M.-H.H.)
4 PhD program in Medical Biotechnology, NCHU, Taichung, Taiwan 40227, R.O.C.; jhliu@nchu.edu.tw (J.-H.L.); mhho@nchu.edu.tw (M.-H.H.)
5 Graduate Institute of Biochemistry, NCHU, Taichung, Taiwan 40227, R.O.C.; jyang@nchu.edu.tw (J.-Y.Y.)
6 Department of Biotechnology, National Kaohsiung Normal University, Kaohsiung, Taiwan 80201, R.O.C.; dwhsu@nknucc.nknu.edu.tw (D.-W.H.)
* Correspondence: jhliu@nchu.edu.tw; Tel.: +886-4-22840338 (J.-H.L.)

**Contents**	**Pages**
Figure S1. Bromide ion-binding sites in XopAI crystals.	S2
Figure S2. Computational prediction of disordered regions in XopAI.	S3
Figure S3. Protein alignment of XopAI homologs.	S4–S5
Figure S4. Protein alignment of XopAI and some known mART proteins.	S6
Figure S5. Structural comparison between XopAI and known mART proteins.	S7
Figure S6. ConSurf analysis for XopAI and HopU1.	S8
Figure S7. Crystal packing of the full-length XopAI and XopAI-ΔN70 proteins.	S9
Figure S8. Intrinsic tryptophan fluorescence studies showing the peptide-binding ability of the XopAI-ΔN70 protein.	S10
Figure S9. Continuous sedimentation coefficient distribution of the full-length XopAI protein.	S11
Figure S10. Comparison of XopAI structures in free and in peptide-bound states.	S12
Figure S11. Comparative plots from MD analysis of the XopAI-peptide complexes	S13
Figure S12. Predicted binding free energy contribution per residue of the protein-peptide interaction during the last 25-ns MD simulation.	S14
Figure S13. Conformational transition between two Arg peptide-binding modes.	S15

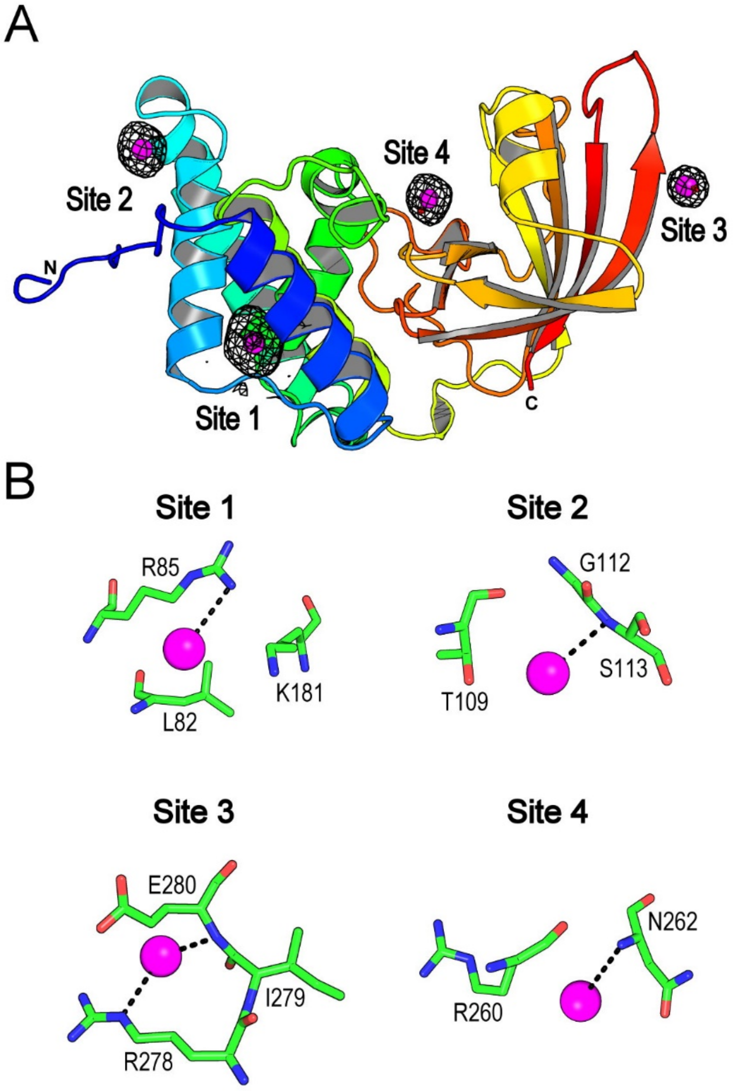

Figure S1. Bromide ion-binding sites in XopAI crystals. **A,** the anomalous difference map (the black mesh) contoured at 2.5 σ that shows four bromide sites (magenta spheres) in the $P4_32_12$ crystal. The XopAI structure is shown as a ribbon model and colored in the rainbow scheme. **B,** close-up views of bromide ion-binding sites. Residues around the bromide ion are shown as stick models and labeled. Carbon, oxygen and nitrogen atoms are colored in green, red and blue, respectively. The potential hydrogen bonds are depicted as black dashed lines.

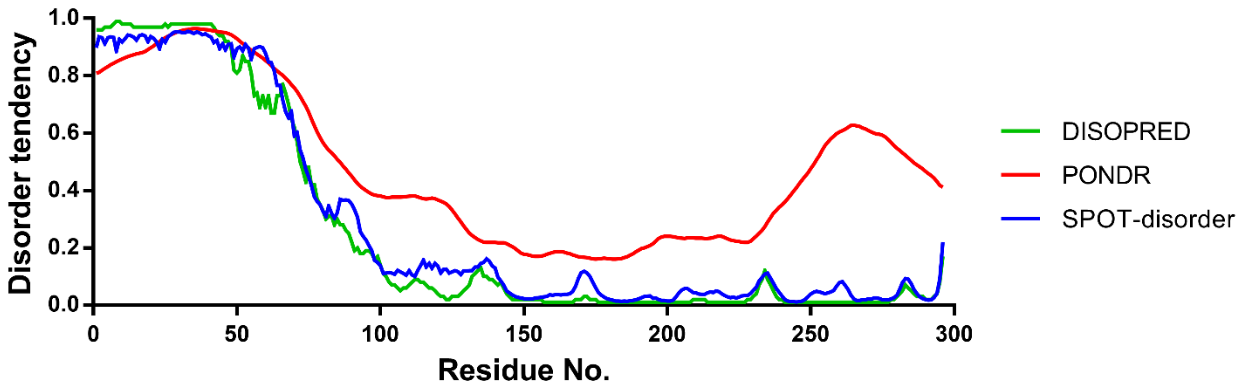

Figure S2. Computational prediction of disordered regions of XopAI. The prediction results were obtained from DISOPRED [40], PONDR [41], and SPOT-disorder [42].

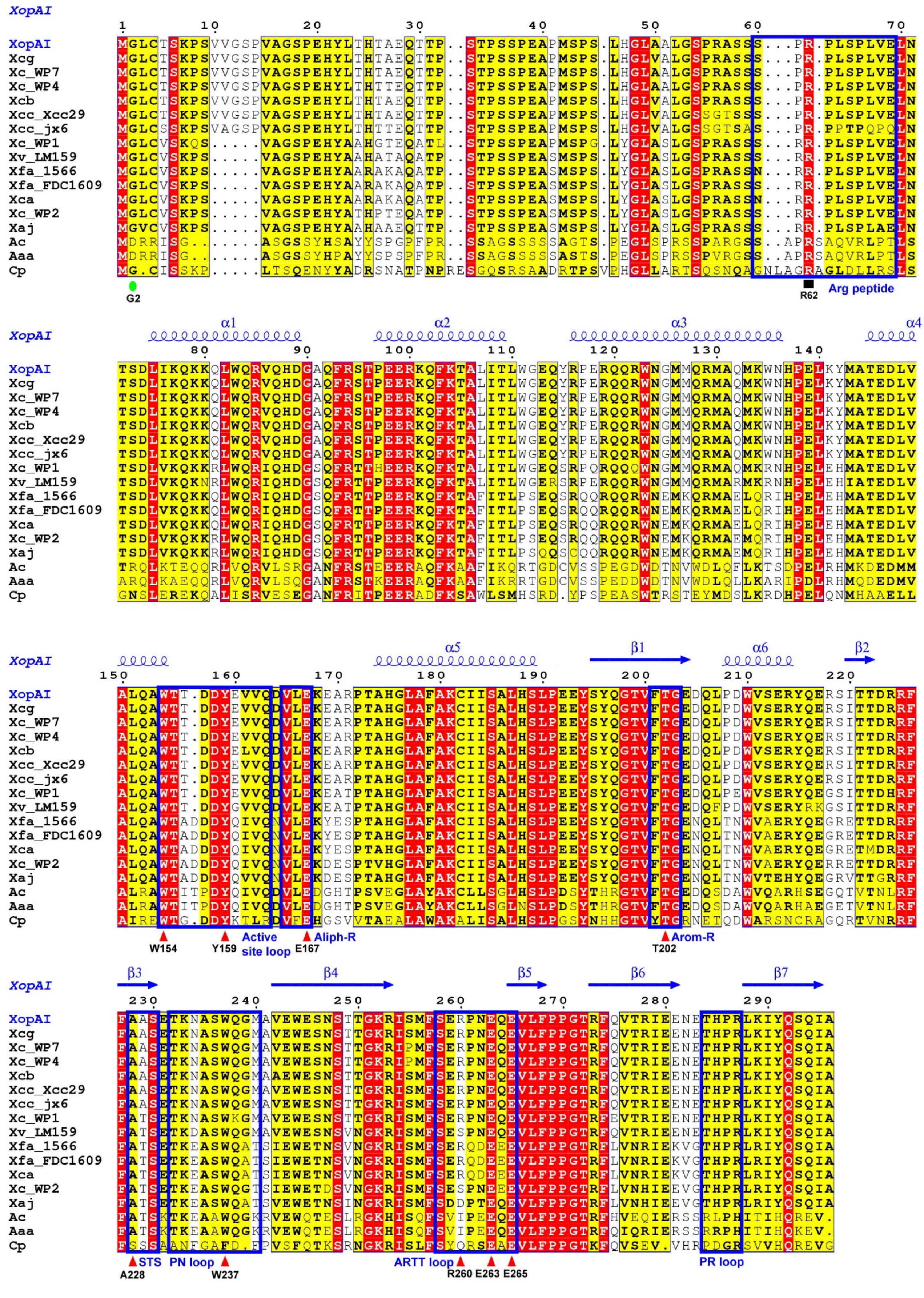

Figure S3. Protein alignment of XopAI homologs. Coloring and labels in this alignment are consistent with

Figure 1C. The following bacteria strains were analyzed: XopAI (*Xanthomonas axonopodis* pv. *citri*, GenBank accession no.: WP_011052119, this study), Xcg (*X. citri* pv. *glycines*, CP017188), Xc_WP7 (*X. citri*, WP_076605129), Xc_WP4 (*X. citri*, WP_040244769), Xcb (*X. citri* pv. *bilvae*, CEJ46851), Xcc_Xcc29 (*X. citri* pv. *citri* strain Xcc29-1, CP023661), Xcc_jx6 (*X. citri* pv. *citri* strain jx-6, CP011827), Xc_WP1 (*X. cynarae*, WP_104591584), Xv_LM159 (*X. vesicatoria* strain LM159, CP018470), Xfa_1566 (*X. fuscans* subsp. *aurantifolii* strain 1566, CP012002), Xfa_FDC1609 (*X. fuscans* subsp. *aurantifolii* strain FDC 1609, CP011163), Xca (*X. citri* pv. *anacardii* CFBP 2913, CP024057), Xc_WP2 (*X. cassava*, WP_029220046), Xaj (*X. arboricola* pv. *juglandis* strain Xaj 417, CP012251), Ac (*Acidovorax citrulli*, WP_011794782), Aaa (*A. avenae* subsp. *avenae*, AVS84630), and Cp (*Collimonas pratensis*, WP_061944107).

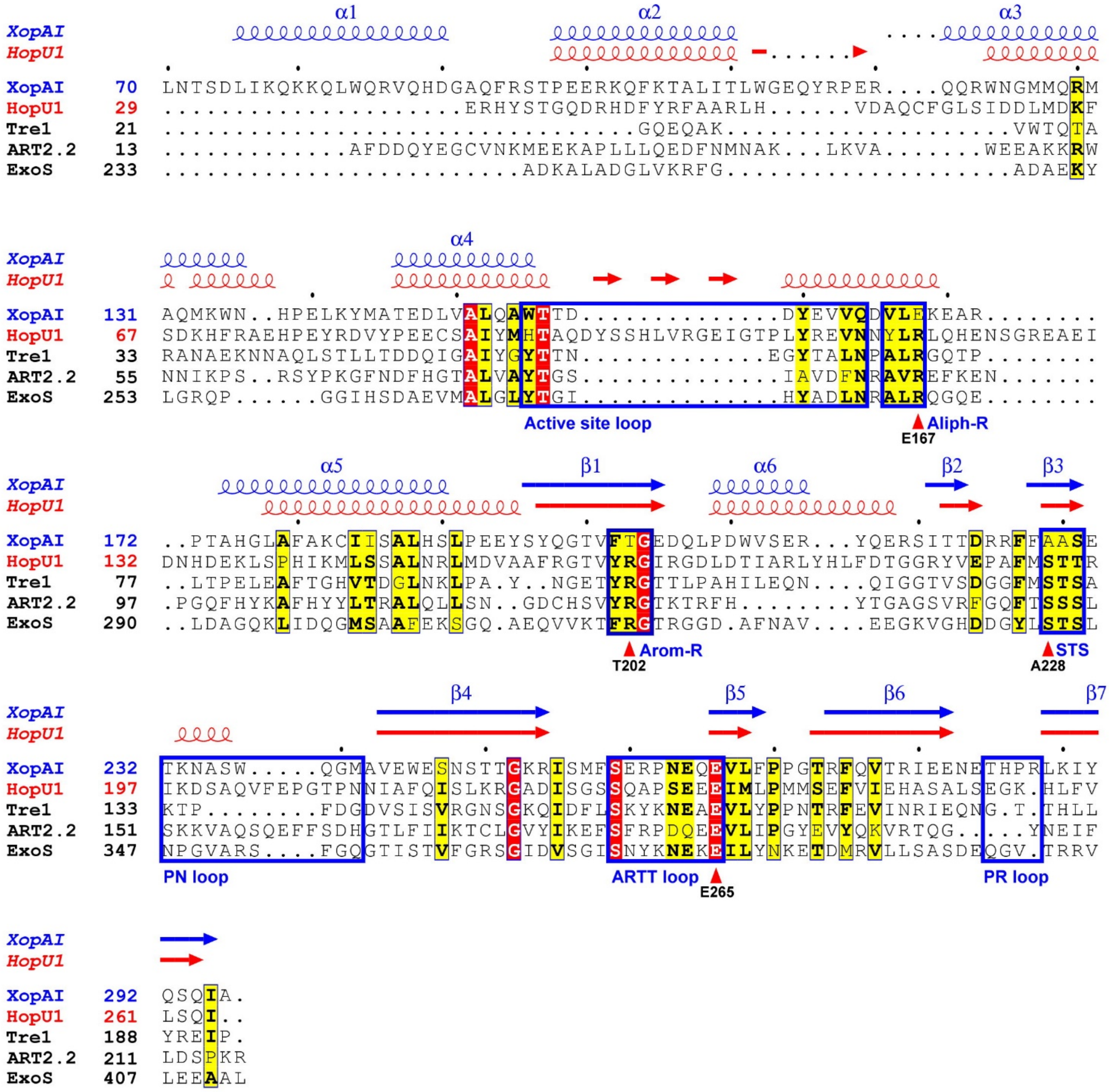

Figure S4. Protein alignment of XopAI and some known mART proteins. Secondary structure elements based on XopAI and HopU1 structures are displayed above the alignment. Blue boxes outline those important regions in mART proteins. The conserved residues in mARTs are marked with red triangles and labeled according to XopAI sequence. The following mART proteins were analyzed: HopU1 (*Pseudomonas syringae* type III-secreted effector HopU1, PDB code 3U0J), Tre1 (*Serratia proteamaculans* type VI secretion ADP-ribosyltransferase effector 1, PDB code 6DRH), ART2.2 (rat mART2.2, PDB code 1GXY), and ExoS (*P. aeruginosa* exoenzyme S, PDB code 6GN8).

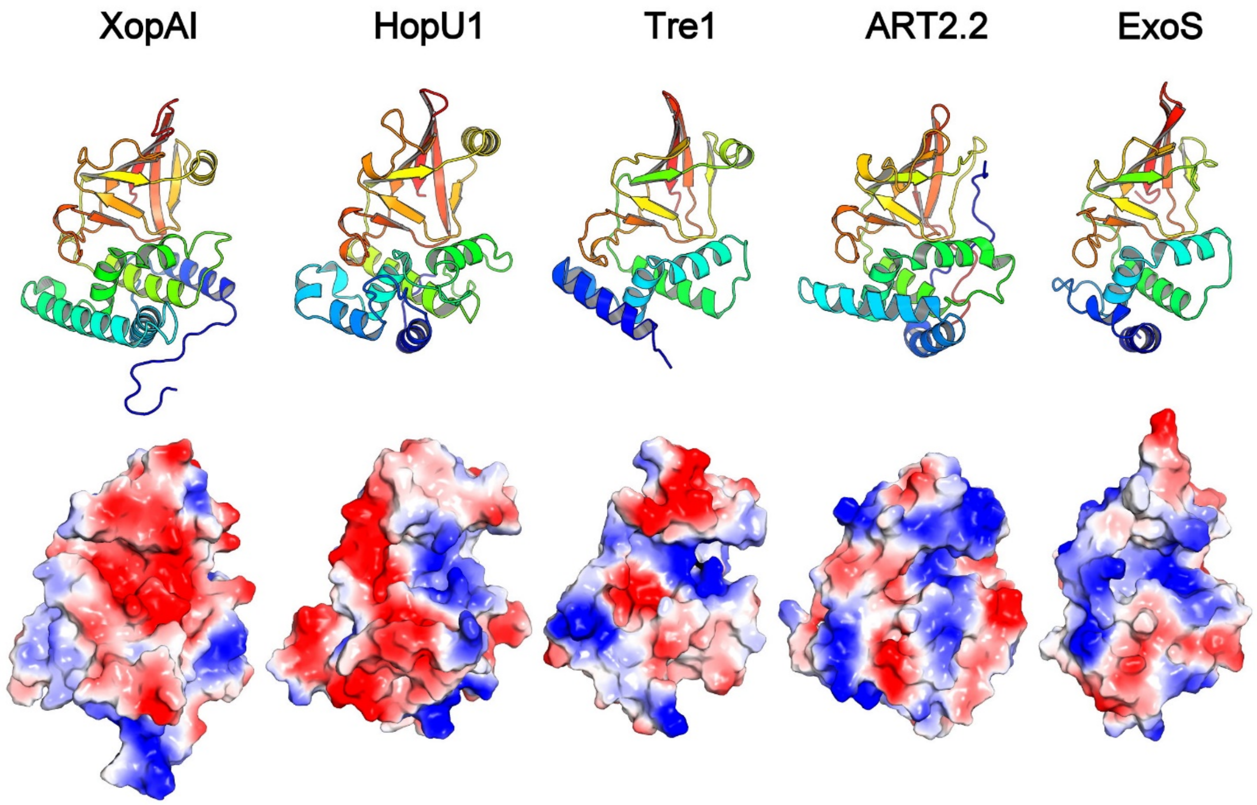

Figure S5. Structural comparison between XopAI and known mART proteins. **A,** ribbon diagram showing XopAI, HopU1 (*Pseudomonas syringae* type III-secreted effector HopU1, PDB code 3U0J), Tre1 (*Serratia proteamaculans* type VI secretion ADP-ribosyltransferase effector 1, PDB code 6DRE), ART2.2 (rat mART2.2, PDB code 1GXY), and ExoS (*Pseudomonas aeruginosa* exoenzyme S, PDB code 6GN8). These structures are colored in the rainbow scheme. **B,** comparison of electrostatic surface potentials. The regions of negative and positive potential are shown in red and blue, respectively; uncharged and hydrophobic surface areas are colorless.

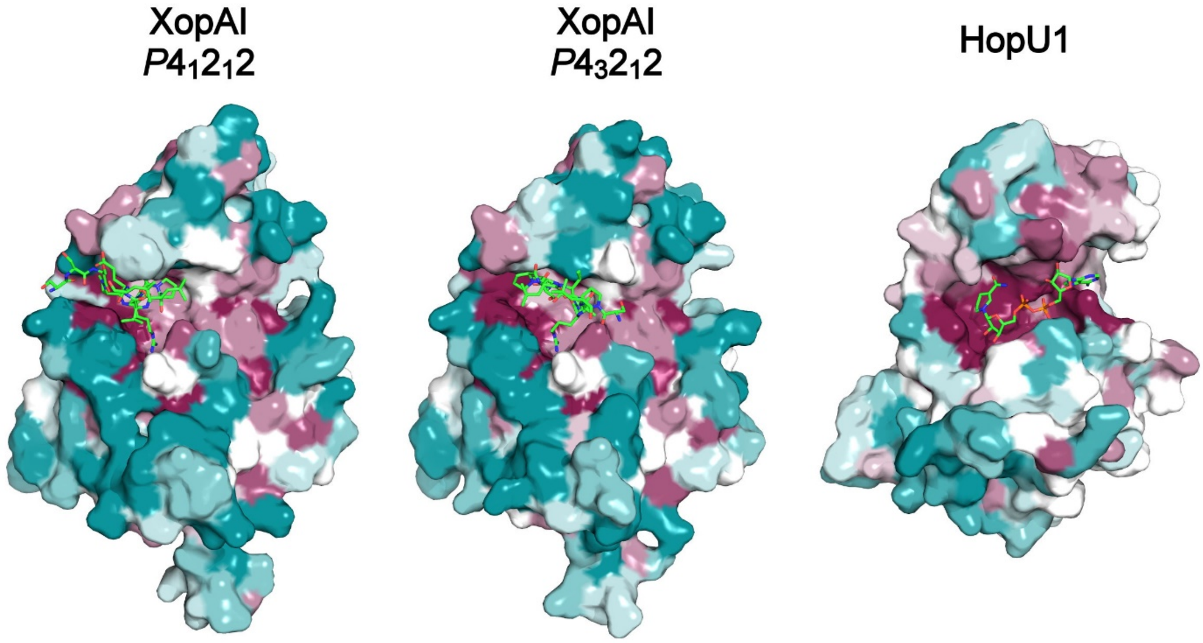

Figure S6. ConSurf analysis for XopAI and HopU1. The surface is color-coded according to the sequence conservation among their homologs, from the most conserved residues (purple) to the least conserved residues (cyan). The Arg peptide bound in XopAI and the cofactor NAD^+ in HopU1 are rendered as stick models. Carbon, oxygen, nitrogen, and phosphorus atoms are colored in green, red, blue, and orange, respectively. The NAD^+ molecule is docked manually into the active site of HopU1 based on the crystal structure of C3stau2 complexed with NAD^+ (PDB code 1OJZ).

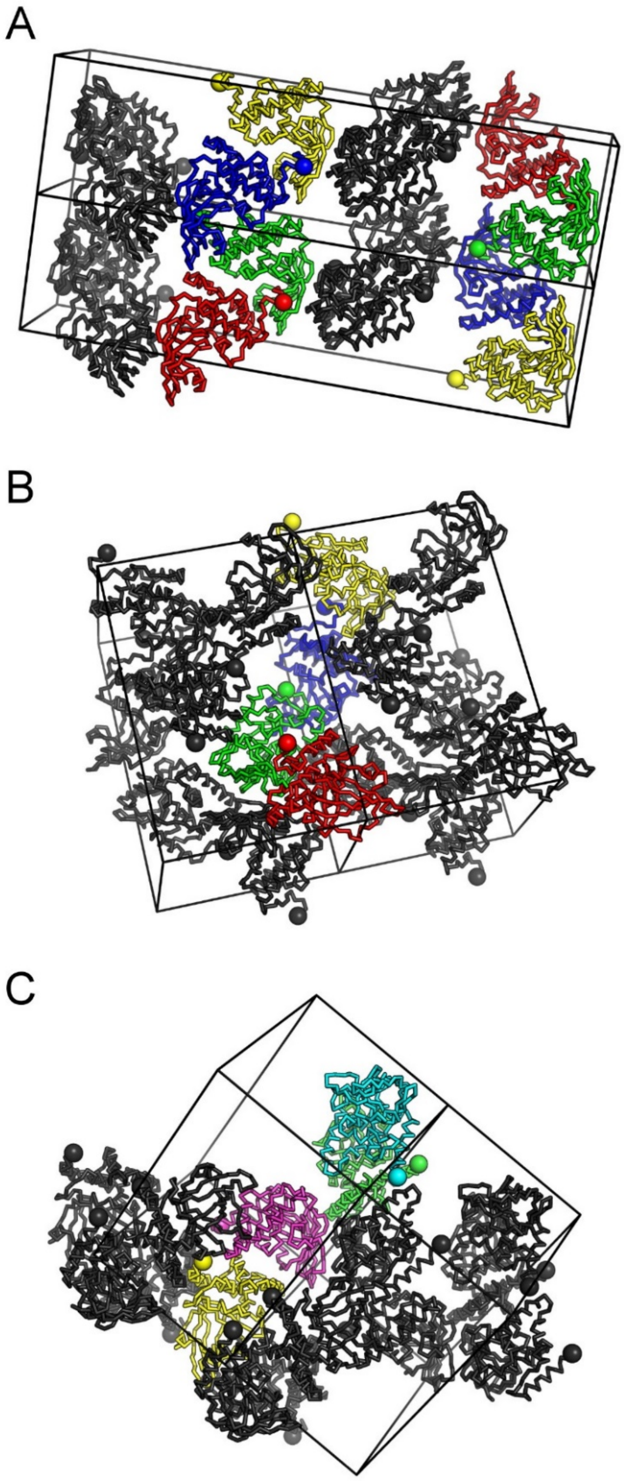

Figure S7. Crystal packing of the full-length XopAI and XopAI-ΔN70 proteins. The crystal packing in a 2 × 1 × 1 supercell is illustrated in the figure. The N-terminus of protein is highlighted as a sphere. **A,** packing of the full-length XopAI in $P4_12_12$ crystals. **B,** packing of the full-length XopAI in $P4_32_12$ crystals. In panels A and B, for clarity, proteins that are packed tandemly are colored in red, green, blue, and yellow, respectively; others are in dark gray. **C,** packing of XopAI-ΔN70 in $P2_1$ crystals. Four proteins in an asymmetric unit are colored in cyan, green, magenta, and yellow, respectively.

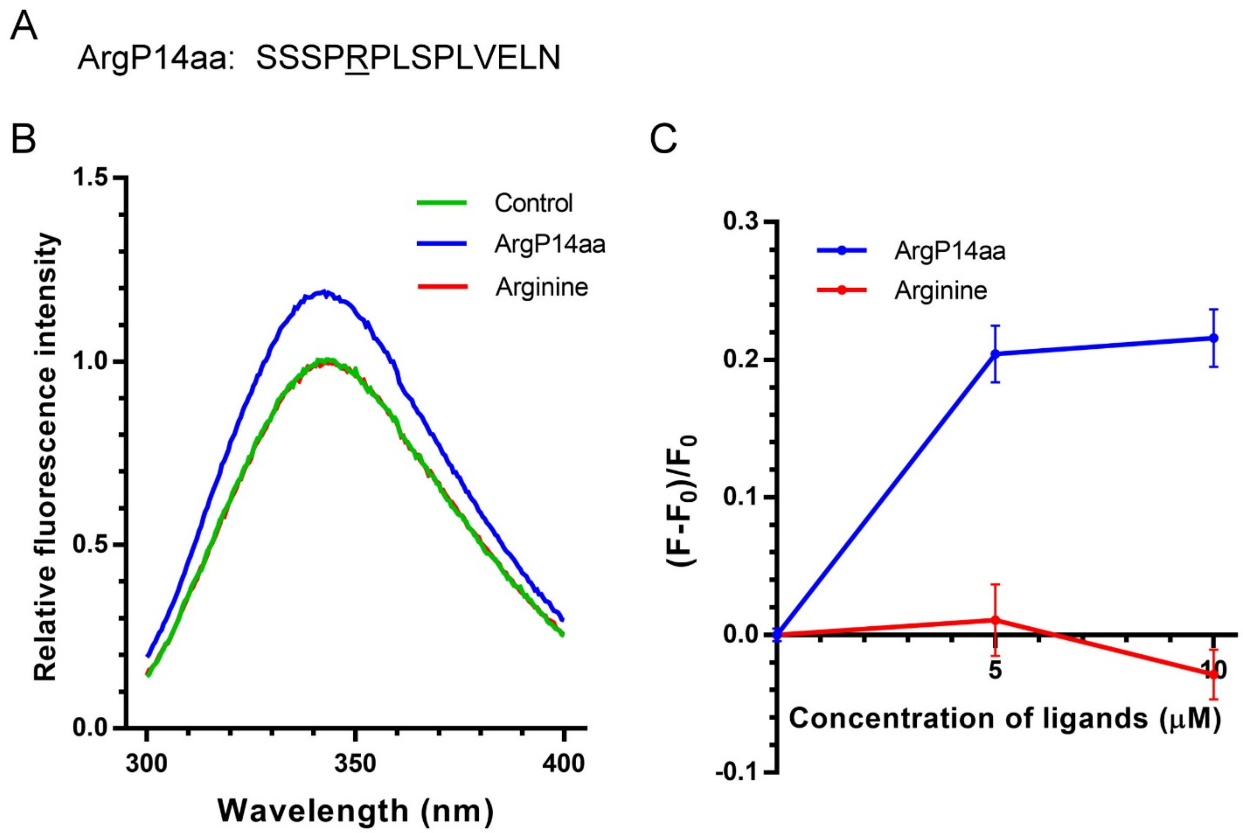

Figure S8. Intrinsic tryptophan fluorescence studies showing the peptide-binding ability of the XopAI-ΔN70 protein. **A,** the sequence of the synthetic peptide ArgP14aa. The position of R62 is underlined. **B,** intrinsic tryptophan fluorescence emission spectra of XopAI-ΔN70 in the absence (green) and presence of ligands (blue and red). The molar ratio of ligand to protein was 1:1. **C,** changes of the emission intensity of XopAI-ΔN70 (5 μM) upon gradual addition of ligands. The y-axis shows the relative change in fluorescence intensity following ligand addition, where F and F_0 are the emission intensities at a certain ligand concentration and in the absence of ligand, respectively. Data points represent means ± standard error (n = 3 separate experiments).

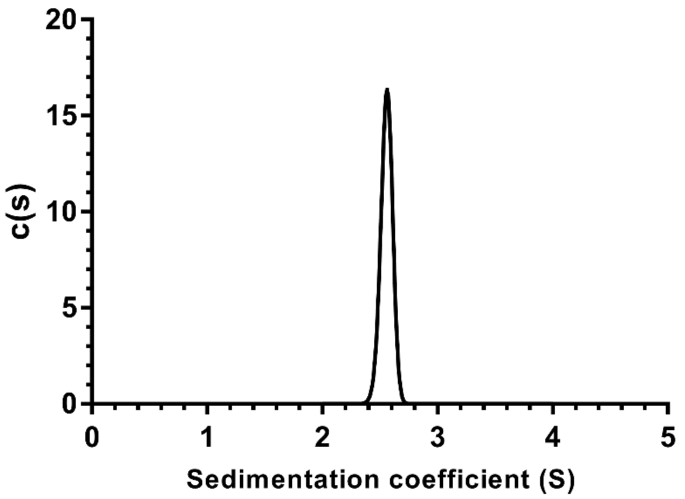

Figure S9. Continuous sedimentation coefficient distribution of the full-length XopAI protein at a concentration of 0.3 mg ml^{-1}. The actual molecular weight of XopAI is 35.8 kDa. The peak having an S value of 2.56 corresponds to a monomeric XopAI.

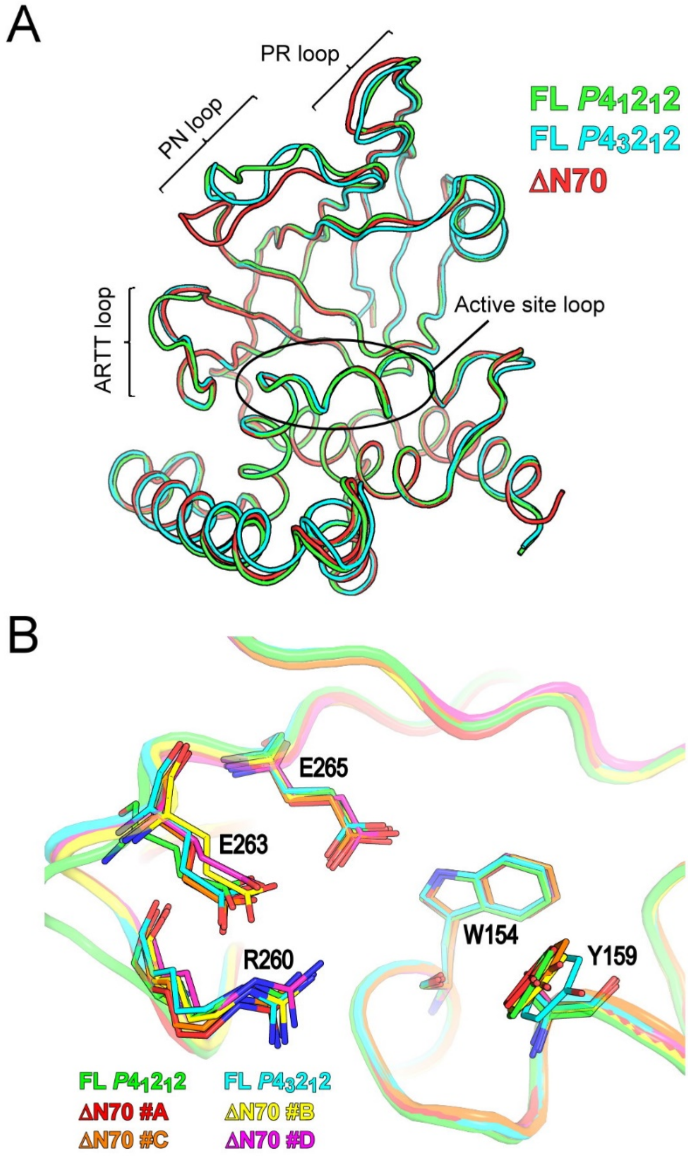

Figure S10. Comparison of XopAI structures in free and in peptide-bound states. **A,** superimposition of XopAI crystal structures. The full-length (FL) proteins are in Arg peptide-bound state, whereas the ΔN70 protein is in an apo state. Protein backbones are colored distinctly as follows: FL $P4_12_12$ (green), FL $P4_32_12$ (cyan), and ΔN70 (red). **B,** superimposition of XopAI peptide-binding pocket in available crystal structures. Protein backbones and carbon atoms are colored distinctly as follows: FL $P4_12_12$ (green), FL $P4_32_12$ (cyan), ΔN70 monomer A (red), ΔN70 monomer B (yellow), ΔN70 monomer C (orange), and ΔN70 monomer D (magenta). Key residues in the peptide-binding cleft are shown as stick models and labeled. Oxygen and nitrogen atoms are colored in red and blue, respectively.

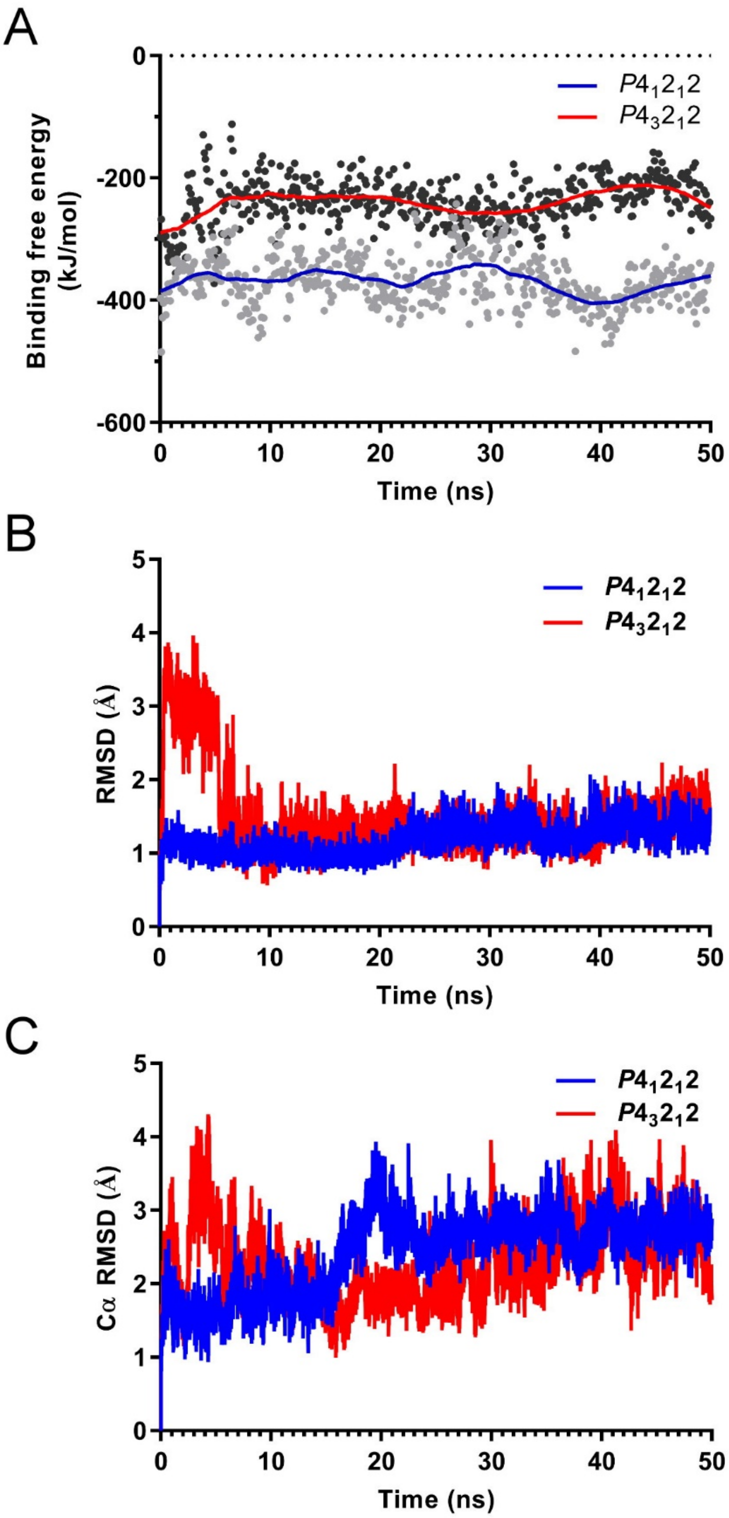

Figure S11. Comparative plots from MD analysis of the XopAI-peptide complexes. **A,** binding energy observed between the peptide-binding cleft and the Arg peptide. **B,** RMSD of all non-hydrogen atoms from R62*. **C,** RMSD of Cα atoms from the Arg peptide.

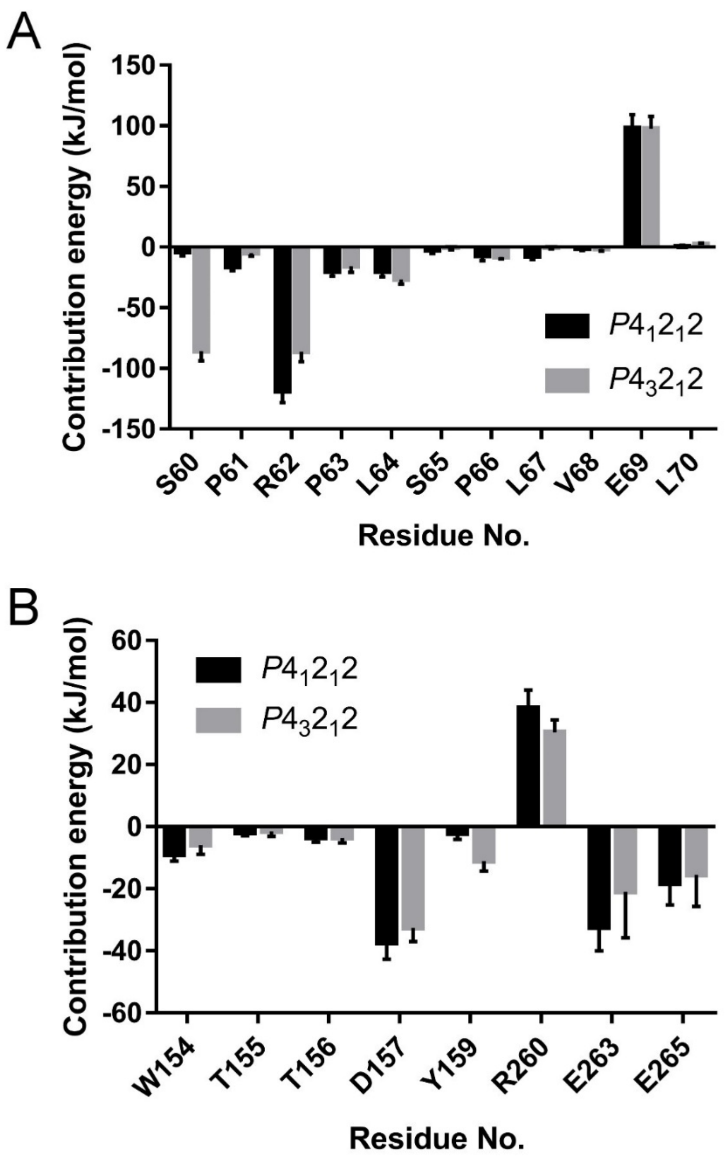

Figure S12. Predicted binding free energy contribution per residue of the protein-peptide interaction during the last 25-ns MD simulation. **A,** the contribution energy of residues in the Arg peptide. **B,** the contribution energy of residues at the Arg-binding site. Please note that the algorithm calculates only the contribution from each amino acid sidechain; a negative value of the contribution energy indicates a positive contribution to the binding.

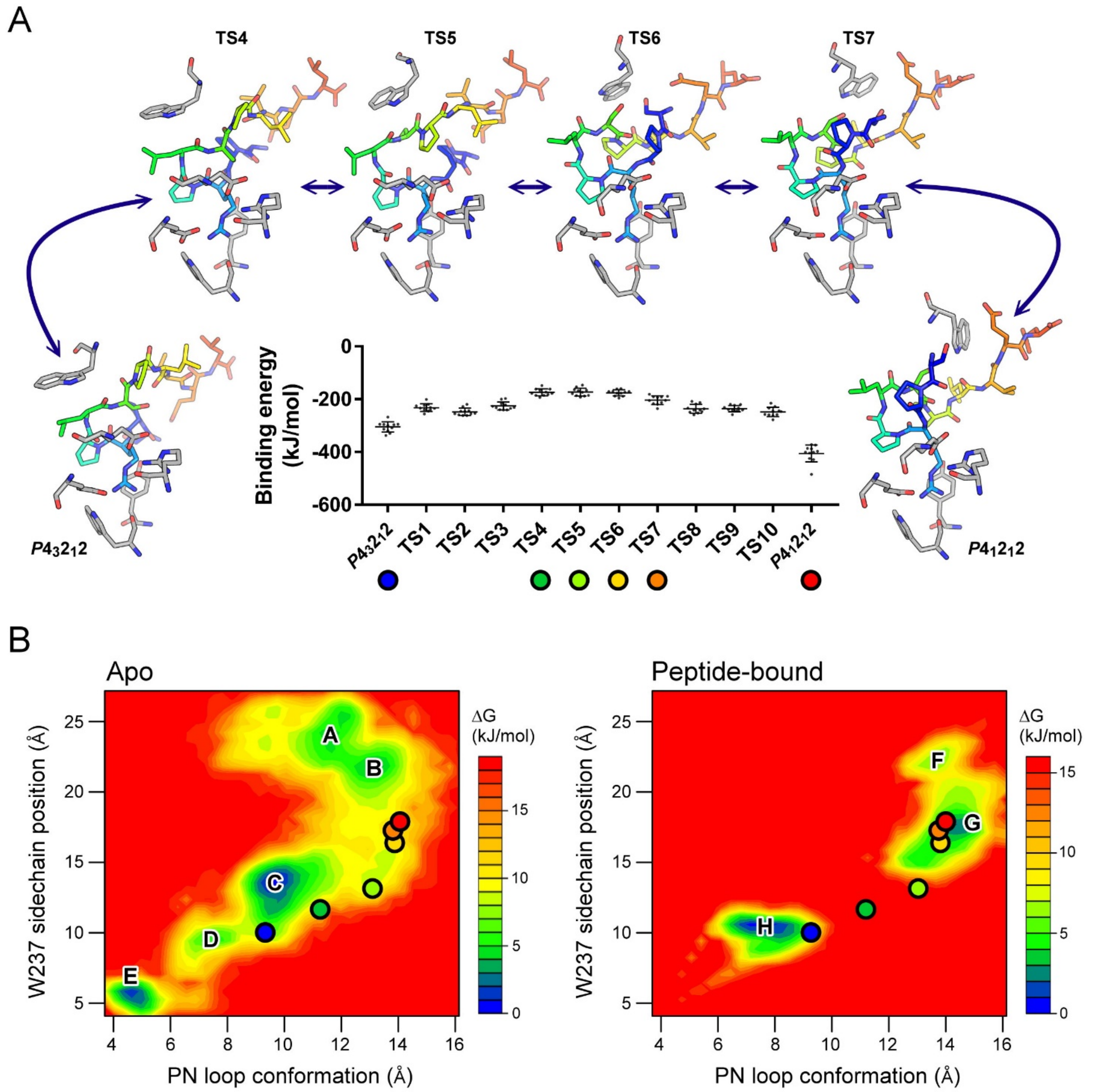

Figure S13. Conformational transition between two Arg peptide-binding modes. **A,** proposed transition states (TSs) between the two observed peptide-binding modes. The scatter plot shows the predicted binding energy of transition states. The selected snapshots (TS4 to TS7) are shown above the scatter plot. The bound Arg peptide in every snapshot is shown as a stick model and colored in the rainbow scheme. Residue W237 and the residues involved in R62* recognition are represented as stick models and their carbons are colored in light gray. The view of snapshots is rotated along the vertical axis by 90° counterclockwise from that of figure 3B. **B,** energy landscapes depicting the motions of the PN loop and residue W237 in the absence (apo) and presence of Arg peptide (peptide-bound). The conformation state of the observed and predicted structures is marked with colored circles, and the color code is indicated at the bottom of scatter plot in panel A.
